# Automated Machine Learning in Dentistry: A Narrative Review of Applications, Challenges, and Future Directions

**DOI:** 10.3390/diagnostics15030273

**Published:** 2025-01-24

**Authors:** Sohaib Shujaat

**Affiliations:** 1 King Abdullah International Medical Research Center, Department of Maxillofacial Surgery and Diagnostic Sciences, College of Dentistry, King Saud Bin Abdulaziz University for Health Sciences, Ministry of National Guard Health Affairs, P.O. Box 3660, Riyadh 11481, Saudi Arabia; sohaib.shujaat941@gmail.com; Tel.: +966-582940293; 2 OMFS IMPATH Research Group, Department of Imaging & Pathology, Faculty of Medicine, KU Leuven & Oral and Maxillofacial Surgery, University Hospitals Leuven, 3000 Leuven, Belgium

**Keywords:** artificial intelligence, automated machine learning, dentistry, oral diagnosis, precision medicine

## Abstract

The adoption of automated machine learning (AutoML) in dentistry is transforming clinical practices by enabling clinicians to harness machine learning (ML) models without requiring extensive technical expertise. This narrative review aims to explore the impact of autoML in dental applications. A comprehensive search of PubMed, Scopus, and Google Scholar was conducted without time and language restrictions. Inclusion criteria focused on studies evaluating autoML applications and performance for dental tasks. Exclusion criteria included non-dental studies, single-case reports, and conference abstracts. This review highlights multiple promising applications of autoML in dentistry. Diagnostic tasks showed high accuracy, such as 95.4% precision in dental implant classification and 92% accuracy in paranasal sinus disease detection. Predictive tasks also demonstrated promise, including 84% accuracy for ICU admissions due to dental infections and 93.9% accuracy in orthodontic extraction predictions. AutoML frameworks like Google Vertex AI and H2O AutoML emerged as key tools for these applications. AutoML shows great promise in transforming dentistry by facilitating data-driven decision-making and improving patient care quality through accessible, automated solutions. Future advancements should focus on enhancing model interpretability, developing large and annotated datasets, and creating pipelines tailored to dental tasks. Educating clinicians on autoML and integrating domain-specific knowledge into automated platforms could further bridge the gap between complex ML technology and practical dental applications.

## 1. Introduction

The integration of machine learning (ML) into dental workflows is advancing clinical decision-making by improving the accuracy and efficiency of diagnostics, treatment planning, and prognosis prediction [1]. Unlike traditional statistical methods, which often rely on linear relationships and predefined models, ML excels at identifying complex, nonlinear interactions, offering a more comprehensive understanding of clinical and radiological dental data [2]. As a result, ML and its deep learning (DL) subset are increasingly being applied across various dental domains, including restorative dentistry, radiology, implantology, periodontology, and oral and maxillofacial surgery. Automated tasks powered by ML mainly include detecting normal anatomy and pathologies, treatment planning, and predicting treatment outcomes [3,4,5].

The widespread adoption of ML in clinical practice remains hindered by technical challenges, particularly in relation to data preprocessing and model development, where an expert oversight is required at each stage. Typically, data scientists or computer engineers are required to handle critical tasks such as data preprocessing, feature extraction, and algorithm selection. Additionally, the fine-tuning of hyperparameters and neural network architecture in the case of DL requires specialized knowledge [6]. For many dentists, this complexity is a significant barrier to adopting ML, as creating and refining effective models demand expertise in data science.

**Figure 1:**
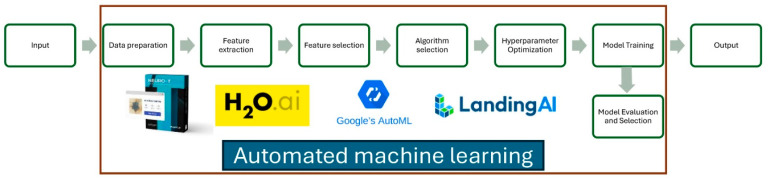
Workflow of automated machine learning where one or more steps can be automated unlike conventional manual machine learning which requires expert oversight.

To address these barriers, automated ML (autoML) has emerged as a practical and innovative solution in the field of artificial intelligence (AI). AutoML encompasses a suite of tools designed to automate the labor-intensive steps of developing, training, and deploying ML models [7]. By removing the need for manual intervention in tasks like data preprocessing, feature selection and engineering, algorithm selection, and hyperparameter tuning, autoML enables non-experts to create high-performing ML models with minimal technical expertise (Figure 1). Platforms such as Google Cloud’s autoML and H2O Driverless AI offer user-friendly interfaces that allow healthcare professionals to experiment with model architectures and algorithms with ease, bridging the gap between advanced ML technology and clinical practice [8].

AutoML has already proven highly effective in various healthcare applications, including medical imaging tasks such as classifying diseases from fundus photographs, segmenting MRI scans, and predicting diabetic retinopathy [9,10,11]. It has also been used to identify COVID-19 carriers and surgical risk prediction [12,13]. AutoML’s scalability and efficiency make it particularly suited for managing the large, complex datasets typical in healthcare settings. By automating key elements of the ML pipeline, autoML accelerates the development and deployment of solutions, reducing reliance on traditional trial-and-error methods for model optimization [14].

While previous reviews have explored the applications of ML and DL across various dental fields, none have specifically explored the role of autoML in dentistry [15,16,17,18]. This narrative review aims to highlight the potential of autoML to streamline workflows, minimize the need for technical expertise, and enhance the precision and reliability in dental applications. It also aims to examine the key challenges, propose future research directions, and explore practical strategies for clinical integration.

## 2. Methodology

This narrative review was conducted to synthesize and evaluate the literature regarding the use of autoML in dentistry. A narrative review approach was chosen to provide a comprehensive overview of the topic, recognizing the heterogeneity of studies and the exploratory nature of autoML applications in dentistry.

Search Strategy: A comprehensive literature search was conducted using databases such as PubMed, Scopus, and Google Scholar. Boolean operators were applied to refine the results. Key search terms included: ((“Automated Machine Learning” OR “AutoML” OR “No-Code AI” OR “Code-Free AI” OR “Low-Code AI”“ OR Automated Deep Learning” OR “AutoDL”) AND (“Dentistry” OR “Oral” OR “Dental Imaging” OR “Diagnostic Dentistry” OR “Predictive Modeling” OR “Risk Assessment” OR “Outcome Prediction”)).

Inclusion and Exclusion Criteria: Studies were included if they focused on the application of autoML in any field of dentistry and provided measurable data on performance metrics for dental tasks. Eligible studies included original research articles, clinical trials, technical reports, brief reports, and case series. No restrictions were placed on language, publication year, or geographic origin to ensure comprehensive coverage. Exclusion criteria were applied to filter out studies that lacked relevance to dental applications, including non-dental fields, non-human studies, single-case reports, preprints, conference abstracts, and editorials.

After the initial screening, full-text reviews of the selected articles were conducted to ensure their relevance and to extract detailed information about methodologies, findings, and implications of autoML applications in dentistry.

## 3. Traditional Models in Dentistry: A Foundation for AI Integration

The evolution of diagnostics and predictive modeling in dentistry began with statistical approaches such as logistic regression and decision trees, which laid the groundwork for more advanced techniques [19,20]. These traditional methods provided early tools for disease diagnosis, risk assessment, and treatment planning, but often required manual feature engineering and assumed linear relationships within data, limiting their ability to handle complex patterns.

The transition to machine learning (ML) methods introduced support vector machines (SVMs) and random forests, which demonstrated high accuracy in identifying dental anomalies, caries, and periodontal diseases [4]. These models improved upon traditional approaches by enabling nonlinear pattern recognition and enhancing decision-making through classification and regression tasks. However, ML approaches require expert oversight for preprocessing, feature selection, and hyperparameter tuning, creating barriers to clinical adoption [4].

Subsequently, DL emerged as a subset of ML, significantly advancing dental imaging tasks by enabling more accurate and efficient image analysis. Convolutional neural networks (CNNs) have become particularly effective for applications such as implant classification, caries detection, and the segmentation of anatomical structures [21]. DL models outperform traditional ML methods by automatically extracting features, thus reducing manual input requirements. Yet, their reliance on large annotated datasets, computational resources, and technical expertise hindered widespread implementation in clinical settings.

While ML and DL have driven advancements, they still face key challenges, including interpretability issues, data quality dependency, and computational overhead. These limitations have prompted the development of autoML, which builds upon the strengths of ML and DL while addressing their limitations. AutoML simplifies model selection, preprocessing, and hyperparameter tuning through automation, reducing the need for technical expertise [22]. Its integration into dental workflows could hold promise for enhancing diagnostic precision, scalability, and interpretability, thus bridging the gap between complex AI methodologies and practical clinical applications. The evidence-based applications of autoML are discussed below.

## 4. Applications of AutoML in Dentistry

AutoML applications in dentistry are still emerging, but they show significant promise in both diagnostic and predictive tasks. No studies were found that used autoML for treatment planning. The following sections explore evidence-based dental applications in which autoML has been successfully implemented to enhance accuracy and efficiency in dental workflows. Table 1 provides a summary of dental tasks utilizing autoML, while Table 2 outlines the platforms used and the level of expertise required to operate each autoML solution effectively.

### 4.1. Diagnostic Tasks

#### 4.1.1. Dental Conditions Classification

Byeon [23] applied autoML in an experimental study for optimizing a convolutional neural network (CNN)-based model called EfficientNet, for panoramic radiograph classification, targeting the automatic detection of conditions such as fillings, implants, and other anomalies. The dataset used consisted of 1272 panoramic radiographs sourced from Kaggle, which were divided into training, validation, and testing sets to evaluate model performance. The author employed the autoML Mobile Neural Architecture Search (MNAS) framework to optimize models for both accuracy and efficiency [38]. They used scaling strategies to balance depth, width, and resolution to enhance model performance while maintaining computational efficiency, which is essential for processing the high-resolution demands of dental radiographs. By automating the model selection, scaling, and hyperparameter tuning, autoML customized EfficientNet’s architecture to meet the specific requirements of the dataset. This approach resulted in a model that effectively managed computational load without compromising diagnostic accuracy. The model demonstrated significant improvements in key performance metrics with an overall accuracy of 90.4%, particularly for underrepresented dental conditions, highlighting autoML’s ability to enhance model robustness.

#### 4.1.2. Paranasal Sinus Disease Classification

Cheong et al. [24] conducted a retrospective study with the application of Google Cloud’s Vertex AI autoML platform (Google LLC, Mountain View, CA, USA) to classify the presence or absence of sinonasal disease using magnetic resonance imaging (MRI) data. The dataset comprised 1383 unique T2-weighted (T2w) non-turbo spin echo (TSE) MRI head sessions from the Open Access Series of Imaging Studies (OASIS-3) repository. Images were labeled by consensus among three specialized head and neck radiologists, and split into 80% training, 10% validation, and 10% testing sets. Data preprocessing included the normalization and exclusion of images with insufficient visual or radiological information. K-fold cross-validation (k = 10) was employed to validate the model. Vertex AI automated several stages of the ML pipeline, including data preparation, model training, hyperparameter tuning, and performance evaluation, thereby simplifying the process and making it accessible to clinicians with limited technical expertise. This study involved training the Vertex AI image classification model using MR scans. After consensus labeling by radiologists, the data were split into training, validation, and testing sets. The model achieved high performance metrics, including 92% accuracy, 91.3% sensitivity, and 92.8% precision. This autoML-driven model highlighted the feasibility of utilizing automated systems to assist radiologists in identifying paranasal sinus diseases, potentially optimizing diagnostic workflows and alleviating the workload of human radiologists.

#### 4.1.3. Dental Implant Classification

Lee et al. [25] conducted an experimental study using Neuro-T version 2.0.1 (Neurocle Inc., Seoul, Republic of Korea), a specialized tool designed to optimize model selection and parameter tuning autonomously. The dataset included 11,980 panoramic and periapical radiographic images collected from three dental hospitals. The images were labeled and classified into six dental implant systems (DISs). The applied tool is designed to automatically select the best model and optimize hyperparameters, settings that influence how well the model learns. By automating these aspects, Neuro-T streamlines the model-building process, removing the need for manual adjustments that typically require advanced knowledge in ML, making it easier to create effective DL models, thereby making sophisticated ML methods more accessible to users without specialized technical expertise. The authors used Neuro-T to implement an automated deep CNN (DCNN) for classifying DISs from panoramic and periapical radiographic images. As a form of autoML, this approach facilitates ML implementation without manual intervention, improving the feasibility of AI technology in clinical applications like dental imaging. Results indicated that the automated DCNN achieved high classification accuracy, surpassing experienced dental professionals in multiple implant classification tasks. The DCNN’s area under the curve (AUC) score was 0.954, reflecting its strong performance in differentiating DIS types. This autoML methodology demonstrates potential as a supportive tool in clinical settings, allowing for efficient and accurate implant identification.

In another experimental study, Kong [26] used Google Cloud’s autoML Vision platform (Google LLC, Mountain View, CA, USA) to classify DISs on periapical images. The autoML Vision model automatically performed neural architecture search and trained a CNN on a dataset of 4800 periapical radiographs belonging to four implant systems (1200 for each implant system). The images were preprocessed, labeled, and split into training, validation, and testing sets automatically. The model achieved high accuracy, with a precision of 0.963 and an overall accuracy of 0.981. This automated approach enabled the development of an accurate implant classification model with minimal manual configuration, making it suitable for clinical applications without requiring advanced programming skills.

#### 4.1.4. Caries and Restoration Detection

Gonzalez et al. [27] developed an autoML-based model in an experimental study to detect primary proximal surface caries in pediatric patients using bitewing radiographs [27]. The dataset consisted of 66 anonymized bitewing radiographs acquired from pediatric dental clinics, meeting inclusion criteria for diagnostic quality and covering primary and mixed dentition stages. Exclusion criteria included artifacts from sensors, positioning errors, and motion distortions. A total of 755 proximal surfaces were identified, of which 178 were annotated as caries lesions by a calibrated expert panel. The platform LandingLens (Landing AI, Palo Alto, CA, USA) was used to develop, train, and evaluate a model without programming requirements, making AI deployment more accessible for clinical use. The model achieved high diagnostic metrics, including an accuracy of 96.4%, and an AUC of 0.965, demonstrating robust performance despite the dataset’s limited size. These findings underscore the potential of no-code AI tools to provide accurate diagnostic support in dentistry, enabling practical applications of computer vision in clinical workflows. In another study, Hamdan et al. [28] performed an experimental study using LandingLens to train a model on panoramic radiographs for dental restoration detection. The dataset comprised 100 anonymized panoramic radiographs selected from electronic patient records at a dental school. A total of 1108 restorations were labeled across 960 teeth within the dataset. The model demonstrated significant effectiveness, achieving sensitivity rates over 86% for pixel-level predictions and over 98% for tooth-level predictions, with an AUC of 0.978.

The autoML approach using LandingLens streamlined several processes that typically require technical expertise, thus allowing dental professionals to develop and implement an advanced ML model without the need for coding skills. The core elements of the autoML process involved automated data partitioning into training, validation, and testing sets, as well as the use of data augmentation methods, such as horizontal and vertical flipping, to enhance model robustness. Furthermore, the configurations of training parameters and model selection were predominantly automated, with users only needing to set basic parameters, like the number of training epochs, while the platform managed more complex optimization tasks. The automated calculation of evaluation metrics—sensitivity, specificity, accuracy, and AUC—enabled the rapid and comprehensive performance assessment of the model. By automating these essential steps, the autoML platform lowered the barriers to AI adoption in dentistry, facilitating the integration of AI-driven diagnostic tools into clinical workflows for practitioners and researchers with limited technical backgrounds.

#### 4.1.5. Gingival Health Classification

Tobias and Spanier [30] conducted a pilot study using autoML-H2O (H2O.ai Inc., Mountain View, CA, USA), a robust supervised learning platform, to classify gingival health based on the Modified Gingival Index (MGI) from dental selfies taken by self-photography. The dataset consisted of 190 dental selfies collected from 44 participants. Each selfie was manually cropped into eight single-tooth images, resulting in a total of 1520 images. MGI scores were assigned by a single board-certified dentist, classifying each image as healthy, mild, or severe inflammation. AutoML-H2O facilitated automated feature selection, model selection, and hyperparameter tuning, streamlining the model-building process and enabling the researchers to focus on feature engineering. This study employed a two-stage cascade of binary classifiers: the first stage classified whether the gums were healthy or inflamed, while the second stage assessed the severity of inflammation. Using autoML-H2O, various algorithms were tested, with XGBoost ML algorithm emerging as one of the most accurate classifiers for this task. In the first classification stage, the model achieved an AUC score as high as 0.99 for specific tooth positions, demonstrating strong accuracy in identifying inflamed gums. In the second stage, which focused on distinguishing between mild and severe inflammation, the model attained AUC scores ranging from 0.84 to 1.0 across different tooth positions on the test set. This highlights the model’s robustness and reliability in accurately classifying varying degrees of gum inflammation based solely on dental selfie images. The autoML approach supported the development of a mobile application called iGAM for the remote monitoring of gum health, which is especially useful for individuals unable to attend in-person consultations.

#### 4.1.6. Root Dental Disease Diagnosis

Jeong [31] demonstrated the utility of Google Vertex AI’s autoML in enabling users regardless of technical expertise to develop and deploy models for dental diagnostics. This experimental study automated hyperparameter tuning and image classification to create a model capable of accurately diagnosing a range of root dental diseases on periapical images. The dataset consisted of 2999 root dental X-ray images obtained from the Kaggle platform. The diseases included various dental anomalies, such as pulpitis, impacted teeth, periodontitis, bone loss, and dental caries, with 95.6% precision and 95.2% recall. This study underscores the transformative power of no-code AI platforms like Google Vertex AI, which allow medical professionals and researchers to innovate more rapidly and inclusively by removing technical barriers. This study highlights the potential for no-code AI in healthcare, suggesting that such platforms can empower medical professionals to enhance diagnostic processes in ways that were previously limited by technical expertise. By simplifying the ML process, Vertex AI enables users to focus more on clinical applications rather than technical complexities. Future developments may include expanding datasets and enhancing autoML’s customization options to further refine diagnostic precision and extend the model’s ability to identify complex root diseases.

#### 4.1.7. Dental Plaque Detection

Nantakeeratipat et al. [29] conducted an experimental study to develop an autoML-based model for detecting dental plaque levels using photographic images of permanent anterior teeth. This study utilized Google Cloud’s Vertex AI autoML platform to automate model training and classification without requiring plaque-disclosing dyes. The dataset consisted of images from 100 dental students, with undyed photographs serving as inputs and erythrosine-dyed images providing ground truth labels categorized into mild (<30%), moderate (30–60%), and heavy (>60%) plaque levels. Two models were developed and evaluated. The first model classified plaque into mild, moderate, and heavy categories with an average precision of 0.907. It performed best for heavy plaque (precision = 0.983) but struggled to distinguish mild and moderate levels due to visual similarities. A simplified binary model, categorizing plaque as acceptable or unacceptable, improved performance with a precision of 0.964 and an F1-score of 0.931. By automating tasks such as data preprocessing, hyperparameter tuning, and model evaluation, autoML enabled the authors to develop scalable and non-invasive solutions without requiring programming expertise. This highlights autoML’s potential for simplifying complex workflows in dental diagnostics.

### 4.2. Prediction Tasks

#### 4.2.1. Intensive Care Unit (ICU) Admission for Dental Infections

Yoon and Park [32] conducted a retrospective study to develop an ML model to predict the need for ICU admission in patients with dental infections using H2O-autoML. The dataset consisted of 210 patient records, and data included demographic characteristics (sex, age, place of residence), systemic factors (hypertension, diabetes mellitus, kidney and liver diseases, heart conditions, anticoagulation therapy, and osteoporosis), and local factors (smoking status, site of infection, postoperative wound infection, dysphagia, odynophagia, and trismus). Initial blood test parameters, such as creatinine, CRP, glucose, hemoglobin, neutrophils, and platelets, were also incorporated. Information on infected fascial spaces (canine, buccal, submental, submandibular, temporal, and deep neck spaces) was added based on contrast-enhanced MDCT imaging. The model was built using only the data available during the initial consultation, including clinical symptoms and blood test results, either alone or in combination with contrast-enhanced multi-detector computed tomography (MDCT) images. The model demonstrated diagnostic accuracy rates of 0.82 using clinical data and blood tests alone, and 0.84 when MDCT images were included. H2O-autoML is particularly well suited for analyzing clinical datasets, offering both classification and regression capabilities. It has the ability to generate high-quality predictive models from tabular data such as patient demographics and clinical test results. Hence, making it a powerful tool for healthcare applications. Furthermore, H2O-autoML’s speed, ease of use, and compatibility with large datasets and multiple programming languages make it an efficient platform for model development and deployment. However, the study did not provide details on the specific settings or parameters used in the autoML process, which needs to be taken into consideration for future studies.

#### 4.2.2. Early Childhood Caries (ECC)

Karhade et al. [33] performed an experimental study using Google Cloud autoML (Google LLC, Mountain View, CA, USA) to predict ECC. The dataset included clinical, demographic, behavioral, and parent-reported oral health status data from 6404 preschool-aged children (mean age = 54 months). ECC cases were defined as one or more decayed, missing, or filled primary surfaces (dmfs) with a threshold based on the International Caries Detection and Assessment System (ICDAS) score ≥ 3. By deploying autoML on Google Cloud, authors efficiently tested various combinations of input variables to assess their impact on the model’s predictive accuracy. The best-performing model achieved an AUC of 0.74 and a sensitivity of 0.67, demonstrating that autoML can generate reliable classifiers for use in community health settings. This model was further validated using external datasets, underscoring autoML’s robustness and its ability to generalize across different populations. This highlights autoML’s potential in healthcare, enabling continuous refinement based on new data from varied sources. Future autoML models for ECC prediction could incorporate additional data, such as genome sequencing or microbiome profiles. Google Cloud was chosen for this study due to its ability to manage large, complex datasets. The platform’s autoML capabilities automate model selection and tuning, making it accessible to researchers without deep ML expertise. Additionally, Google Cloud provides robust data storage, real-time performance monitoring, and collaborative tools, enhancing the efficiency and impact of predictive modeling in pediatric dental health research.

#### 4.2.3. Orthodontic Extraction

Real et al. [34] retrospectively assessed the effectiveness of autoML in generating predictive models for orthodontic extractions using Auto-WEKA version 2.0 (University of British Columbia, Vancouver, BC, Canada), a user-friendly autoML system. The dataset consisted of 214 anonymized orthodontic cases extracted from a clinical database of 314 patients. The dataset incorporated seven clinical variables and seventeen cephalometric variables, including overjet, overbite, maxillary and mandibular arch discrepancies, molar and canine class, as well as sagittal and vertical skeletal measurements like ANB, SNA, SNB, and Rickett’s facial depth. The system automatically selected and optimized relevant features from patient data such as maxillary and mandibular discrepancies and cephalometric molar relationships. It then performed model selection by evaluating multiple ML algorithms, followed by hyperparameter tuning to maximize accuracy. Using nested cross-validation, Auto-WEKA ensured model robustness by repeatedly training and testing the models, thus reducing overfitting. The results revealed that autoML could produce highly accurate predictive models, with an accuracy of 93.9%. This highlights the potential of autoML to simplify model creation and optimization, making it a valuable tool for orthodontists in clinical decision-making.

#### 4.2.4. Metabolic Syndrome in Periodontal Disease Patients

Boitor et al. [35] conducted a retrospective study to develop and compare autoML-based models to predict metabolic syndrome in patients with periodontal disease, utilizing the H2O autoML and Auto-sklearn 2.0 (University of Freiburg, Freiburg, Germany) frameworks. The dataset consisted of 296 patients (aged 45–79 years). Clinical and periodontal data were collected, including the Decayed, Missing, and Filled Teeth (DMFT) index, Community Periodontal Index (CPI), periodontal pocket depth, gingival attachment loss, gingival bleeding, daily tooth brushing habits, dental control frequency, cardiovascular risk, carotid atherosclerosis, and EuroQol 5-Dimensions 5-Levels (EQ-5D-5L) scores. Patients were classified based on metabolic syndrome criteria outlined by the National Cholesterol Education Program Adult Treatment Panel III (NCEP ATP III). Both frameworks simplified and automated essential ML steps, including algorithm selection, hyperparameter tuning, and model training, which are typically resource-intensive and require significant expertise. By automating these steps, the frameworks minimized manual intervention, making the process more efficient. H2O-autoML’s approach had a comprehensive set of algorithms, including advanced ensemble methods that combined predictions from multiple models to boost accuracy. Auto-sklearn, built on scikit-learn, offered a robust model selection process using Bayesian optimization, designed to identify the best-performing models within specific computational and time constraints. The findings indicated that H2O-autoML (100%) outperformed Auto-sklearn (95.5%), achieving perfect classification accuracy. This result highlights the potential of autoML, particularly H2O-autoML, in building highly accurate predictive models for complex clinical applications.

#### 4.2.5. Medication-Related Osteonecrosis of the Jaw (MRONJ) Risk

Kwack and Park [36] conducted a retrospective study using H2O-autoML to predict medication-related osteonecrosis of the jaw (MRONJ) in osteoporosis patients who had undergone tooth extractions or dental implant placements. The dataset consisted of 340 female patients aged 55 years or older. Demographic, systemic, and local factors were analyzed, including age, hypertension, hyperlipidemia, diabetes, medication duration, the number of operated teeth, and operation area (maxilla, mandible, or both). Patients were divided into two groups—control (*n* = 210) and MRONJ (*n* = 130). The authors applied autoML to automate key steps such as algorithm selection and hyperparameter optimization for the model-building process. Instead of manually testing and tuning various algorithms, H2O-autoML efficiently evaluated all algorithms and their respective hyperparameters, identifying the best-performing model. This automated approach selected the Gradient Boosting Machine (GBM) algorithm as the most appropriate one for the task at hand. The GBM model performed well during training (AUC = 0.8283), and this strong performance was largely maintained during testing (AUC = 0.7526), indicating good model generalization. The study provides valuable insights into the use of autoML for MRONJ prediction, underscoring the importance of factors such as medication duration, age, and other clinical variables in risk assessment.

#### 4.2.6. Age Prediction

Xu et al. [37] performed an experimental study using Google Cloud autoML to develop an age prediction model based on facial images. The dataset consisted of 4908 facial images of Asian individuals, sourced from the open access MegaAge dataset. The images were categorized into seven age groups: below 10, 10–20, 20–30, 30–40, 40–50, 50–60, and above 60. The authors uploaded a dataset of facial images based on different age groups to Google autoML for training. The autoML platform automated tasks such as data splitting, model training, and hyperparameter optimization, resulting in an initial model with a precision of 64.89%. By adjusting the confidence threshold to 0.71, the precision was improved to 69.83%. This approach allowed for efficient model development without the need for extensive programming. The study demonstrated that autoML can facilitate the development of ML models, achieving satisfactory precision for age categorization based on facial features.

## 5. Evidence Synthesis

Across the reviewed studies, consistent patterns and gaps emerged regarding the applications of autoML in dentistry. AutoML frameworks demonstrated exceptional utility in streamlining workflows, automating hyperparameter tuning, and achieving high diagnostic and predictive accuracy, particularly in image-based diagnostic tasks. Studies such as those by Byeon et al. [23] and Hamdan et al. [28] demonstrated strong performance metrics for image classification and restoration detection. However, there was a distinct gap in evaluating autoML for non-imaging tasks, such as treatment planning, monitoring disease progression, and prognosis modeling.

Additionally, while some studies successfully applied autoML to structured clinical datasets for predictions [32,35], challenges remain regarding dataset standardization, interpretability, and external validation. The reliance on small and retrospective datasets raises concerns about overfitting and the ability of these models to generalize to larger, more diverse populations.

Studies focusing on structured data [32,35] demonstrated the suitability of H2O-autoML for clinical datasets, particularly for tabular data, due to its ability to handle classification and regression tasks effectively. In contrast, frameworks like Google AutoML Vision excelled in image-based workflows [23], showcasing their strength in tasks requiring visual pattern recognition. This highlights the need for framework-specific evaluations tailored to different data types in dentistry. For instance, H2O-autoML proved advantageous for analyzing structured datasets, while Google AutoML Vision demonstrated superior performance in image classification tasks. At the same instance, Real et al. [34] identified scalability limitations in the Auto-WEKA framework, particularly when dealing with large datasets and computationally intensive processes, emphasizing the importance of selecting frameworks based on data size and task complexity.

Another common trend observed was the preference for frameworks like H2O-autoML [30,32,35,36] and those using the Google platform [24,26,29,31,33,37] due to their ease of use and scalability. However, limitations such as reduced interpretability and cloud dependence on infrastructure were highlighted [28,32,35]. Framework-specific challenges, such as scalability issues in Auto-WEKA [34] and reduced customization in Google AutoML Vision [26], suggest the need for adaptable, transparent AI solutions to enhance clinical usability.

Despite the promising results, no studies evaluated the long-term clinical impact of autoML implementations, emphasizing the need for prospective trials and real-world validation. Many studies relied on retrospective data, limiting insights into how these frameworks perform in real-time clinical workflows. Reporting inconsistencies in metrics [34,37], complicate cross-study comparisons due to variations in performance measures like AUC, accuracy, and sensitivity. Furthermore, frameworks optimized for scalability and interpretability require thorough testing in clinical environments to assess their usability, transparency, and ability to handle large-scale datasets.

Addressing these gaps will be critical for ensuring the reliable integration of autoML into routine dental practice. Given the reliance on retrospective data and feasibility studies, future research must prioritize prospective trials and real-time clinical validation to establish long-term performance and reliability. Additionally, integrating longitudinal datasets could enable the monitoring of disease progression, bridging gaps identified in current research. The evidence base remains fragmented, underscoring the necessity of harmonizing evaluation metrics and establishing standardized protocols for testing autoML frameworks.

## 6. Limitations and Challenges

### 6.1. General Limitations of AutoML Frameworks

Table 3 provides a summary of autoML frameworks used in the studies with their strengths and weaknesses.

In general, a significant challenge of applying autoML in dental healthcare is its “black box” nature, which limits transparency in model decision-making [7]. While autoML’s design simplifies complex ML tasks, enabling non-experts to engage with predictive modeling, this also reduces the interpretability of how the model arrives at its outputs. In clinical settings, where decisions affect patient care, this lack of clarity may reduce clinicians’ trust in the models [8]. Consequently, the absence of insight into a model’s rationale can be a barrier to its adoption, especially in scenarios with high clinical impact.

Another challenge lies in autoML’s dependence on extensive, high-quality datasets, which are often scarce in oral healthcare [2]. Effective autoML models require well-annotated and comprehensive data to achieve accuracy and generalization. However, in dentistry, data are often limited, inconsistent, or lack diversity, especially for rare conditions, which may impair model performance and restrict its applicability across diverse populations. Additionally, while autoML minimizes the technical workload, clinicians must still ensure data accuracy, oversee feature selection, and monitor models to maintain clinical relevance.

AutoML’s computational demands also pose practical limitations. Developing accurate models, especially those processing high-resolution images, requires significant computational power, which may not be feasible for smaller clinical practices [4]. Furthermore, when working with dental datasets, autoML must adhere to frameworks such as the Health Insurance Portability and Accountability Act (HIPAA) in the United States and the General Data Protection Regulation (GDPR) in the European Union. As autoML platforms often provide limited transparency and control compared to manual ML workflows, this might potentially influence the pace of its adoption in clinical settings.

### 6.2. Critique of the Included Literature

The studies included in this review exhibited considerable variation in their methodologies, sample sizes, and data preprocessing techniques, reflecting the heterogeneity inherent in autoML applications within dentistry. While most studies demonstrated high accuracy, their retrospective or experimental nature limits the generalizability of findings. For instance, some studies relied on small datasets [27,32,34], which raises concerns regarding overfitting and external validity when applied to broader populations. Moreover, studies predominantly focused on image-based tasks, leaving gaps in the exploration of autoML for non-imaging applications, such as treatment planning and prognosis modeling.

A key limitation observed in the evidence hierarchy was the lack of large-scale multi-center studies or prospective evaluations to assess autoML performance in real-world clinical workflows. Most studies relied on retrospective data or experimental setups, which limit their ability to demonstrate generalizability and clinical impact. Additionally, the standardization of evaluation metrics and reporting formats varied, making direct comparisons across studies challenging.

### 6.3. Strengths and Limitations of the Review

The main strength of this narrative review is the broad overview of autoML applications in dentistry; however, it is not without limitations. Being conducted by a single author, there is a potential for selection bias in the choice of included studies and reporting bias in highlighting favorable outcomes over limitations. Furthermore, it inherently lacks the systematic rigor and reproducibility associated with systematic reviews or meta-analyses, which may limit replicability.

Heterogeneity in study design, sample sizes, and frameworks used further complicates drawing unified conclusions about autoML’s performance across diverse dental tasks. While attempts were made to mitigate this by categorizing studies into diagnostic and predictive tasks, the interpretative nature of the review leaves room for subjective bias. Future systematic reviews could address these issues through collaborative authorship, structured methodologies, and the inclusion of meta-analyses to improve reliability and reproducibility.

## 7. Future Directions

Due to the limited evidence on the use of autoML for dental applications, future research should focus on rigorously evaluating its applicability and performance across various dental tasks. Comparative studies are also encouraged to assess how autoML-driven outcomes measure up against those from traditional ML models [39], examining aspects such as accuracy, efficiency, and clinical relevance. Such investigations will help establish guidelines for effectively integrating autoML in dentistry and potentially pave the way for more automated, accessible, and accurate diagnostic, planning, and predictive tools.

Improving model interpretability by incorporating more transparent algorithms or integrating explainable AI tools (XAI) with autoML systems can enhance interpretability, helping clinicians understand the model’s decision-making process [40]. This will be especially crucial in high-stakes scenarios like oral cancer detection and treatment planning. Additionally, enabling compliance with regulatory bodies and encouraging clinical adoption will be crucial [35].

Developing large, diverse, and annotated dental datasets can significantly enhance autoML’s ability to automatically identify important features, optimize model selection, and adapt learning across various dental tasks, with minimal manual intervention.

Educational initiatives aimed at familiarizing clinicians with autoML applications in dentistry could further enhance adoption by reducing reliance on data scientists. These efforts would empower dental professionals to leverage autoML frameworks directly, enabling the development of clinically relevant AI tools without requiring extensive technical expertise. Large language models (LLMs), such as ChatGPT, could also assist clinicians in enhancing autoML workflows by providing support for coding tasks, data formatting, and explaining modeling processes [41]. However, while LLMs offer valuable assistance, their outputs must be validated using external methodologies to ensure accuracy, compliance, and clinical reliability before integration into autoML workflows.

General autoML solutions may not be optimized for the specific requirements of dental image processing or structured data. Developing customized pipelines tailored to dental clinical and imaging datasets could be beneficial. Current autoML tools lack an understanding of specific dental domain knowledge, such as the anatomical structure of teeth or the patterns of dental diseases [42]. Creating domain-specific models or embedding dental ontologies into autoML tools may further improve their accuracy. For example, models could be pre-trained with a basic understanding of dental anatomy, which would allow them to identify features in images more accurately.

Developing autoML systems that can utilize longitudinal datasets to track changes and predict disease progression is essential [43]. Such systems would be valuable for monitoring chronic conditions like periodontal disease or assessing post-surgical healing in implant cases.

Dental imaging frequently involves 3D data, such as cone-beam CT scans, intraoral scans and facial images. Developing autoML capabilities for 3D imaging to support tasks like the 3D segmentation of teeth and bone structures or assessing complex pathologies could allow the further adoption of autoML in orthodontics, implant planning, and maxillofacial surgery.

## 8. Conclusions

This narrative review has highlighted the potential of autoML for automating diagnostic and predictive workflows in dentistry, enabling clinicians to utilize AI models with minimal technical expertise. While autoML can simplify processes and improve accuracy, it is not a replacement for data scientists but rather a complementary tool to enhance collaboration between clinicians and AI experts. This review has examined key challenges, including issues related to model interpretability, data quality, and computational demands, and has explored actionable recommendations such as the development of standardized datasets, improved transparency, and clinician-focused training programs. By providing insights into these areas, this review has established a foundation for future research to validate autoML’s performance across diverse clinical contexts and support its integration into real-world practice.

## Figures and Tables

**Table 1:** Summary of applications using automatic machine learning (autoML).

Reference	Dental Specialty	Study Type	Main Task	Category	AutoML Framework	Performance
Byeon [23]	Radiology	Experimental study	Panoramic radiographic classification	Diagnostic	AutoML Mobile Neural Architecture Search (EfficientNet)	Area under the curve: approximately 0.95
Cheong et al. [24]	Radiology	Retrospective feasibility study	Paranasal sinus disease classification	Diagnostic	Google Vertex AI	92% accuracy, 91.3% sensitivity
Lee et al. [25]	Implantology	Experimental study	Dental implant classification	Diagnostic	Neuro-T	Area under the curve: 0.95, precision: 0.96
Kong [26]	Implantology	Experimental study	Dental implant classification	Diagnostic	Google AutoML Vision	Accuracy: 0.98
Gonzalez et al. [27]	Restorative Dentistry	Experimental study	Caries detection	Diagnostic	LandingLens	Precision: 95.8%, accuracy: 96.4%, Area under the curve: 0.96
Hamdan et al. [28]	Restorative Dentistry	Experimental study	Dental restoration detection	Diagnostic	LandingLens	Accuracy: 99.63%, F1-score: 0.844 by pixel; accuracy: 98.21%, AUC: 0.978
Nantakeeratipat et al. [29]	Periodontology	Experimental study	Dental plaque detection	Diagnostic	Google Vertex AI	Precision: 0.964, F1-score: 0.931
Tobias and Spanier [30]	Periodontology	Pilot study	Gingival health classification	Diagnostic	H2O AutoML	Area under the curve: up to 1.0
Jeong [31]	Endodontics	Experimental study	Root dental disease diagnosis	Diagnostic	Google Vertex AI	Area under the curve: 0.96, precision: 95.6%
Yoon and Park [32]	Oral and Maxillofacial Surgery	Retrospective study	Intensive care unit admission for dental infections	Prediction	H2O AutoML	Area under the curve: 0.84
Karhade et al. [33]	Restorative Dentistry	Experimental study	Early childhood caries	Prediction	Google Cloud AutoML	AUC: 0.74
Real et al. [34]	Orthodontics	Retrospective study	Orthodontic extraction	Prediction	Auto-WEKA	93.9% accuracy
Boitor et al. [35]	Periodontology	Retrospective study	Metabolic syndrome in periodontal disease patients	Prediction	H2O AutoML, Auto-sklearn	H2O AutoML: 100% accuracy, Autosklearn: 95.5% accuracy
Kwack and Park [36]	Oral and Maxillofacial Surgery	Retrospective study	Medication-related osteonecrosis of the jaw risk prediction	Prediction	H2O AutoML	Area under the curve: 0.75
Xu et al. [37]	General Dentistry	Experimental study	Age prediction	Prediction	Google Cloud AutoML	Precision improved to 69.83%

**Table 2:** Automatic machine learning (autoML) frameworks and level of expertise required.

AutoML Framework	Code Requirement Level	Links
AutoML MNAS (EfficientNet) [23]	Code Required	https://github.com/tensorflow/tpu/tree/master/models/official/efficientnet (accessed on 16 December 2024)
Google Vertex AI [24,29,31]	No Code	https://cloud.google.com/vertex-ai (accessed on 16 December 2024)
Neuro-T [25]	Low-Code	https://www.neuro-cle.com/en (accessed on 16 December 2024)
Google AutoML Vision [26]	No Code	https://cloud.google.com/vision/automl (accessed on 16 December 2024)
H2O-AutoML [30,32,35,36]	Low Code	https://www.h2o.ai/products/h2o-automl/ (accessed on 16 December 2024)
Google Cloud AutoML [33,37]	No Code	https://cloud.google.com/automl (accessed on 16 December 2024)
Auto-WEKA [34]	Low Code	https://www.cs.ubc.ca/labs/beta/Projects/autoweka/ (accessed on 16 December 2024)
Auto-sklearn [35]	Code Required	https://automl.github.io/auto-sklearn/master/ (accessed on 16 December 2024)
LandingLens [27,28]	No Code	https://landing.ai/platform (accessed on 16 December 2024)

Note: No Code: ideal for clinicians or researchers with limited or without coding skills, Low Code: require minimal coding, primarily for setup or parameter adjustments, Code Required: require programming and are generally more suited to technical users.

**Table 3:** Strengths and weaknesses of applied automated machine learning (automL) frameworks in dentistry.

AutoML Framework	Method	Strengths	Weaknesses
AutoML MNAS (EfficientNet) [23]	Neural architecture search for deep learning models, automated optimization.	- Efficient model architectures optimized for image tasks. - Good computational efficiency for large datasets. - High accuracy in image classification tasks.	- Requires advanced knowledge to fine-tune for specific problems. - Computationally expensive and requires substantial resources.
Google Vertex AI [24,29,31]	No-code, cloud-based model for machine learning.	- Accessible for non-experts. - Scalable for large datasets. - Integration with Google Cloud ecosystem.	- Limited flexibility for advanced users. - Performance depends on data quality.
Neuro-T [25]	Automated deep learning for specialized tasks.	- High precision in specific tasks like implant classification. - Automated hyperparameter tuning for optimization.	- Limited flexibility for broader tasks. - Needs preprocessing adjustments.
Google AutoML Vision [26]	No-code interface for image-based tasks.	- Quick deployment for image-based tasks. - User-friendly, especially for clinicians. - Integration with Google services.	- Limited customization for hyperparameter tuning. - Best suited for standard tasks and less flexible for complex models.
H2O-autoML [30,32,35,36]	Automated machine learning platform with focus on model selection and training.	- Minimal code required. - Supports both classification and regression tasks. - Great for ensemble learning and improving model accuracy.	- May struggle with complex tasks requiring deep customization. - Slower performance with very large datasets.
Google Cloud AutoML [33,37]	No-code platform for model creation that integrates seamlessly with Google Cloud services.	- Extremely user-friendly and great for non-technical users. - Cloud-based, so it supports scalable applications. - Fast deployment for standard tasks.	- Limited customization and hyperparameter tuning. - May not perform well for complex, non-standard tasks.
Auto-WEKA [34]	Bayesian optimization used for automated machine learning to select models, hyperparameters, and preprocessing strategies.	- Highly customizable and well suited for researchers. - Excellent for automating model selection and feature engineering.	- Not suitable for large datasets. - Can be slow with big data and requires parameter tuning.
Auto-sklearn [35]	Automated machine learning with model selection and feature engineering based on traditional machine learning algorithms.	- Highly flexible for advanced users. - Efficient feature selection. - Great for complex research tasks.	- Requires coding expertise, and the setup can be time-consuming. - Best suited for researchers, not clinicians.
LandingLens [27,28]	No-code platform optimized for vision-specific tasks.	- Fast deployment for computer vision tasks. - Designed specifically for clinician use, making it easy to interpret results. - Optimized for detection and segmentation tasks.	- Struggles with noisy or complex data. - May not handle very large datasets as efficiently as other frameworks.
